# Inhibition of iNOS as a novel effective targeted therapy against triple-negative breast cancer

**DOI:** 10.1186/s13058-015-0527-x

**Published:** 2015-02-22

**Authors:** Sergio Granados-Principal, Yi Liu, Maria L Guevara, Elvin Blanco, Dong Soon Choi, Wei Qian, Tejal Patel, Angel A Rodriguez, Joseph Cusimano, Heidi L Weiss, Hong Zhao, Melissa D Landis, Bhuvanesh Dave, Steven S Gross, Jenny C Chang

**Affiliations:** Methodist Cancer Center, Houston Methodist Hospital, 6445 Main Street, P21-34, Houston, TX 77030 USA; Medical and Health Sciences College, Monterrey Institute of Technology, Eugenio Garza Sada 2501, Monterrey, NL 64849 Mexico; Department of Nanomedicine, Houston Methodist Research Institute, 6670 Bertner Avenue, Houston, TX 77030 USA; Department of Chemistry and Biochemistry, Arizona State University, 551 E University Dr., Tempe, AZ 85287 USA; Biostatistics Shared Resource Facility, Markey Cancer Center, University of Kentucky, 800 Rose Street, Lexington, KY 40536-0093 USA; Department of Systems Medicine and Bioengineering, Houston Methodist Research Institute, 6670 Bertner Avenue, Houston, TX 77030 USA; Weill Cornell Medical College, 1300 York Avenue, New York, NY 10065 USA

## Abstract

**Introduction:**

Triple-negative breast cancer (TNBC) is an aggressive form of breast cancer with no effective targeted therapy. Inducible nitric oxide synthase (iNOS) is associated with poor survival in patients with breast cancer by increasing tumor aggressiveness. This work aimed to investigate the potential of iNOS inhibitors as a targeted therapy for TNBC. We hypothesized that inhibition of endogenous iNOS would decrease TNBC aggressiveness by reducing tumor initiation and metastasis through modulation of epithelial-mesenchymal transition (EMT)-inducing factors.

**Methods:**

iNOS protein levels were determined in 83 human TNBC tissues and correlated with clinical outcome. Proliferation, mammosphere-forming efficiency, migration, and EMT transcription factors were assessed *in vitro* after iNOS inhibition. Endogenous iNOS targeting was evaluated as a potential therapy in TNBC mouse models.

**Results:**

High endogenous iNOS expression was associated with worse prognosis in patients with TNBC by gene expression as well as immunohistochemical analysis. Selective iNOS (1400 W) and pan-NOS (L-NMMA and L-NAME) inhibitors diminished cell proliferation, cancer stem cell self-renewal, and cell migration *in vitro*, together with inhibition of EMT transcription factors (Snail, Slug, Twist1, and Zeb1). Impairment of hypoxia-inducible factor 1α, endoplasmic reticulum stress (IRE1α/XBP1), and the crosstalk between activating transcription factor 3/activating transcription factor 4 and transforming growth factor β was observed. iNOS inhibition significantly reduced tumor growth, the number of lung metastases, tumor initiation, and self-renewal.

**Conclusions:**

Considering the effectiveness of L-NMMA in decreasing tumor growth and enhancing survival rate in TNBC, we propose a targeted therapeutic clinical trial by re-purposing the pan-NOS inhibitor L-NMMA, which has been extensively investigated for cardiogenic shock as an anti-cancer therapeutic.

**Electronic supplementary material:**

The online version of this article (doi:10.1186/s13058-015-0527-x) contains supplementary material, which is available to authorized users.

## Introduction

Triple-negative breast cancer (TNBC) is an aggressive and lethal form of cancer that lacks estrogen receptor alpha (ERα), progesterone, and human epidermal growth factor receptors with no approved targeted therapeutic options. Despite numerous advances, treatment resistance and metastasis are the main causes of death in patients with TNBC. Resistance to conventional treatment and onset of metastases may arise from a subpopulation of cancer stem cells (CSCs) with self-renewal and tumor-initiating capacities [1,2]. Thus, combinatorial treatments with conventional chemotherapy and anti-CSC therapies would be necessary to reduce tumor burden, recurrence, and metastasis to distant organs [3,4].

Nitric oxide (NO) is a bioactive molecule that exhibits pleotropic effects within cancer cells and tumors, with concentration-dependent pro- and anti-tumor effects. NO is produced by three different nitric oxide synthase (NOS) isoforms: neuronal (nNOS/*NOS1*), inducible (iNOS/*NOS2*), and endothelial (eNOS/*NOS3*) [5]. Increased iNOS expression has been found in breast cancer [6-9] and other different cancers such as lung [10], colon [11], melanoma [12], and glioblastoma [13]. Previous reports have demonstrated a correlation between high iNOS expression, aggressiveness, and poor prognosis in patients with breast cancer [6-9]. Increased iNOS expression has recently been postulated as a prognostic factor for reduced survival in patients with basal-like ERα-negative breast cancer through the induction of interleukin-8 (*IL-8*), *CD44*, *c-Myc* [7] and partially due to the activation of the transcription factor Ets-1 [14]. Here, we hypothesize that enhanced endogenous iNOS expression drives poor patient survival by promoting tumor relapse and metastases through modulation of CSC self-renewal properties and tumor cell migration. We further hypothesize that, in combination with conventional chemotherapy, the inhibition of endogenous iNOS would reduce the aggressiveness of residual TNBC cells and mesenchymal features and the number of metastases to distant organs, thus improving survival of patients with TNBC. We studied the inhibition of iNOS with different small-molecule inhibitors: the selective iNOS inhibitor 1400 W and two pan-NOS inhibitors: L-NMMA and L-NAME. L-NMMA has been extensively studied in hundreds of patients for cardiogenic shock [15] and, if efficacious, would enable immediate translation into clinical trials without the need of extensive preclinical testing.

## Methods

### Reagents

N-[[3-(aminomethyl)phenyl]methyl]-ethanimidamide (1400 W) and N5-[imino(nitroamino)methyl]-L-ornithine methyl ester (L-NAME) were purchased from Cayman Chemical (Ann Arbor, MI, USA). Tilarginine (N^G^-Monomethyl-L-arginine) (L-NMMA) was from Santa Cruz Biotechnology (Dallas, TX, USA) and kindly supplied by (Arginox Pharmaceuticals, Redwood City, CA, USA). Tunicamycin and recombinant human TGF-β1 were obtained from Abcam (Cambridge, UK) and PeproTech (Rocky Hill, NJ, USA), respectively. iNOS (N-20), eNOS (C-20), nNOS (R-20), Twist1 (L-21), Twist1 (2C1a), ATF3 (C-19), and CREB-2 (C-20) antibodies were from Santa Cruz Biotechnology. Antibodies Snail (C15D3), Slug (C19G7), TCF8/Zeb1 (D80D3), PERK (C33E10), TGFβ, phospho-Smad2/3 (D6G10), Smad2/3, IRE1α (14C10), phospho-PERK (16 F8), PERK (C33E10), phospho-eIF2α (119A11), eIF2α, β-Actin, anti-rabbit, and anti-mouse IgG were obtained from Cell Signaling Technology (Danvers, MA, USA). Hypoxia-inducible factor 1α (HIF1α) (EP1215Y) was from Abcam.

### Oncomine gene expression data analysis

Relative levels of *NOS2* mRNA expression in human TNBC were investigated by Oncomine Cancer Microarray database analysis [16] of The Cancer Genome Atlas (TCGA) database (n = 593). Patient survival analysis of two different gene expression data sets was obtained [17,18].

### Cell culture

Mesenchymal-like TNBC cell lines MDA-MB-231 and SUM159 were purchased from American Type Culture Collection (Manassas, VA, USA) and Asterand Bioscience (Detroit, MI, USA), respectively. These cell lines were chosen on the basis of their high expression of epithelial-mesenchymal transition (EMT) markers, metastatic properties, percentage of CD44^+^/CD24^−^ cells, iNOS protein levels, similar protein levels of iNOS downstream targets, and similar production of total NO (data not shown). Cells were grown in Dulbecco’s modified Eagle’s medium (DMEM) (Gibco, Life Technologies, Grand Island, NY 14072 USA) supplemented with 10% fetal bovine serum and 1% antibiotic-antimycotic. Unless otherwise specified, cells were treated daily with 1400 W (0.1, 1, 10, 100 μM; 1, 2, 4 mM), L-NMMA (0.1, 1, 10, 100 μM; 1, 2, 4 mM), or L-NAME (0.1, 1, 10, 100 μM; 1, 2, 5 mM) for 96 hours.

For mammosphere (MS) formation (MSFE), cells were cultured for 96 hours under treatment in 0.5% methylcellulose and MammoCult basal medium (StemCell Technologies, Vancouver, BC, Canada) supplemented with 10% proliferation supplements, 4 μg/mL heparin, and 0.48 μg/mL hydrocortisone. Primary MSs were scanned and counted with GelCount (Oxford Optronix, Abingdon, UK). Secondary MSs were grown in the absence of treatment. For the mouse model of lung metastasis, MDA-MB-231 cells were transfected with a luciferase/GFP-based dual-reporter plasmid and stable clones (MDA-MB-231 L/G) selected with blasticidin (InvivoGen, San Diego, CA, USA).

### Cell proliferation assay

Proliferation of SUM159 and MDA-MB-231 was determined by adding premixed WST-1 reagent (Clontech, Mountain View, CA, USA). For transient knockdown in SUM159 and MDA-MB-231 cells (500 cells per well), proliferation was determined after 72 hours of transfection.

### Wound healing assay

Confluent cells were treated in starvation conditions (1% serum) for 72 hours. Medium was changed by regular growth medium in the presence of inhibitors for 24 hours more. For transient knockdown, cells were transfected for 72 hours in growth media. A ‘wound’ was then created in the cell monolayer with a 100-μL pipette tip. Images were taken at 0 and 14 hours. Data were replicated in three independent experiments.

### RNA interference experiments

SUM159 and MDA-MB-231 cells were transiently transfected with Scrambled siRNA, siRNA1, or siRNA2 (100 nM) (Silencer Select; Ambion, Life Technologies, Grand Island, NY 14072 USA) for 96 hours using Lipofectamine RNAiMAX (Invitrogen, Life Technologies, Grand Island, NY 14072 USA) in accordance with the instructions of the manufacturer. GIPZ lentiviral *NOS2* (shRNA1 - V3LHS_360691) and empty vector shRNAs were purchased from Thermo Fisher Scientific. MDA-MB-231 cells were transduced with lentiviral particles and selected with puromycin. Cells were then harvested and plated for immunocytochemistry of iNOS.

### Nitric oxide production

Cells were treated with L-NMMA or 1400 W for 24 hours in phenol red- and serum-free DMEM. Aliquots of cell culture supernatant were taken at 0, 0.5, 2, 6, and 24 hours for total NO production with the nitrate/nitrite fluorometric assay kit (Cayman Chemical) in accordance with the instructions of the manufacturer.

### Western blot

Whole cell lysates were made in 1X lysis buffer (Cell Signaling Technology) and 1X protease/phosphatase inhibitor cocktail (Thermo Scientific). Samples (30 μg protein) were boiled in sample buffer (Thermo Scientific) containing β-mercaptoethanol (Sigma-Aldrich, St. Louis, MO, USA) and subjected to SDS-PAGE electrophoresis in 4% to 20% polyacrylamide gels (Bio-Rad, Hercules, CA, USA). Proteins were transferred onto nitrocellulose membranes (Bio-Rad). Membranes were incubated overnight at 4°C with primary antibodies (1:1,000) and the appropriate secondary antibodies for 1 hour (1:2,000). Protein bands were developed in autoradiography films (Denville Scientific Inc., South Plainfield, NJ, USA).

### RT-PCR analysis of spliced XBP1

cDNA was synthetized from total RNA and subsequently amplified by polymerase chain reaction (PCR). The primers were *XBP1* (5′-GGGTCCAAGTTGTCCAGAATGC-3′ and 5′-TTACGAGAGAAAACTCATGGC-3′) and β-Actin (5′-CTGGAACGGTGAAGGTGACA-3′ and 5′-AAGGGACTTCCTGTAACAATGCA-3′). PCR conditions were 1 cycle at 95°C for 5 minutes, 25 cycles of 30 seconds at 95°C, 1 minute at 50°C, and 1 minute at 68°C, followed by 1 cycle at 68°C for 5 minutes. cDNA amplicons were resolved in 2% agarose.

### Immunohistochemistry

After antigen retrieval (Tris-Cl, pH 9.0), paraffin-embedded sections of human patient samples and xenograft tumors were incubated for 1 hour at room temperature with iNOS (1:50) (Novus Biologicals, Littleton, CO, USA), Ki67 (1:100) (Abcam), and cleaved caspase-3 (1:50) (Cell Signaling Technology) antibodies. The iNOS score method was as follows: intensity (0 to 3): negative, weak, moderate, strong; distribution (0 to 4): <10%, 10% to 30%, >30% to 50%, >50% to 80%, >80%. Total score can be divided into four groups: negative (0 or 1), weak (2 or 3), moderate (4 or 5), and strong (6 or 7) as previously reported [7]. MDA-MB-231 cells transfected either with *NOS2*-directed shRNA (shRNA1) or empty vector were used as negative and positive control of iNOS staining, respectively.

### Animal studies

Either MDA-MB-231 or SUM159 cells (3 × 10^6^) were injected in the right mammary fat pad of female severe combined immunodeficiency (SCID) Beige mice. Once the tumors reached 150 to 200 mm^3^, the mice were randomly assigned as follows (n = 10 per group): (1) vehicle (saline, intraperitoneal, or i.p.), (2) L-NMMA (either 80 mg/kg or 200 mg/kg, i.p., daily), (3) docetaxel (20 mg/kg), and (4) combo (L-NMMA and docetaxel). For the lung metastases-preventing study, MDA-MB-231 L/G cells were implanted as described above. The mice were randomly assigned, and treatments started 48 hours after cell injection (n = 5 per group): (1) vehicle (saline, i.p.) and (2) L-NAME (80 mg/kg, i.p., daily for 35 days). Lungs were removed and incubated in cold complete DMEM containing 50 μM luciferin for 10 minutes. Luminescent cancer cells were detected with an IVIS-200 *in vivo* imaging system (PerkinElmer, Waltham, MA, USA). The clinically relevant dose regimen consisted on two cycles of docetaxel (20 mg/kg, i.p., on day 0), L-NMMA (400 mg/kg on day 1, and 200 mg/kg for 4 additional days by oral gavage), and amlodipine on day 0 (10 mg/kg, i.p., daily, for 6 days). Docetaxel alone as well as saline (i.p.) + sterile water (oral gavage) were used as controls.

MS formation and limiting dilution assay (LDA) were assayed as previously described [4]. All animal procedures and experimental protocols were approved by the Houston Methodist Research Institute Animal Care and Use Review Office that ensured adherence to the National Institutes of Health Guide for the Care and Use of Laboratory Animals.

### Metabolite profiling by liquid chromatography-tandem mass spectrometry

Xenograft tissue as well as plasma samples were prepared as previously described [19]. L-NMMA (200 mg/kg) was orally administered by gavage to female SCID Beige mice (n = 5). Blood was drawn before (baseline, 0 hours) and after (0.5, 2, 12, and 24 hours) L-NMMA administration. Ratiometric quantification of methylarginine (L-NMMA) and citrulline was determined as ion abundance levels in plasma and tumor tissue [19].

### Blood pressure

Blood pressure (BP) was measured in 15 female SCID Beige mice for 3 days (basal BP) and subsequently treated with one cycle of the clinically relevant dose regimen (n = 5 per group). The average daily BP was determined by averaging the last 10 of 20 BP measurements for the last three consecutive days of the cycle treatment using a computerized tail cuff monitor (BP-2000 Series II; Visitech Systems, Napa Place, Apex, NC, USA).

### Statistical analysis

All data were analyzed by using GraphPad Prism (GraphPad Software, La Jolla, CA, USA). Data are presented as mean ± standard error of the mean. Statistical significance between two groups was analyzed by two-tailed Student’s *t* test. Experiments with more than three groups were analyzed with one-way analysis of variance (ANOVA) and Bonferroni’s *post hoc* test. Statistical analysis of tumor volume was assessed by two-way ANOVA and Bonferroni’s *post hoc* test. Fisher’s exact test was used to determine significant differences in LDA. Survival proportions were assessed by using a Kaplan-Meier method and further analyzed with either Wilcoxon or log-rank test. Proliferation, MSFE, migration index, and Ki67 staining are normalized to the vehicle group (100%). A *P* value of less than 0.05 was considered significant.

## Results

### Enhanced iNOS expression correlates with poor patient survival in invasive TNBC

**Figure 1:**
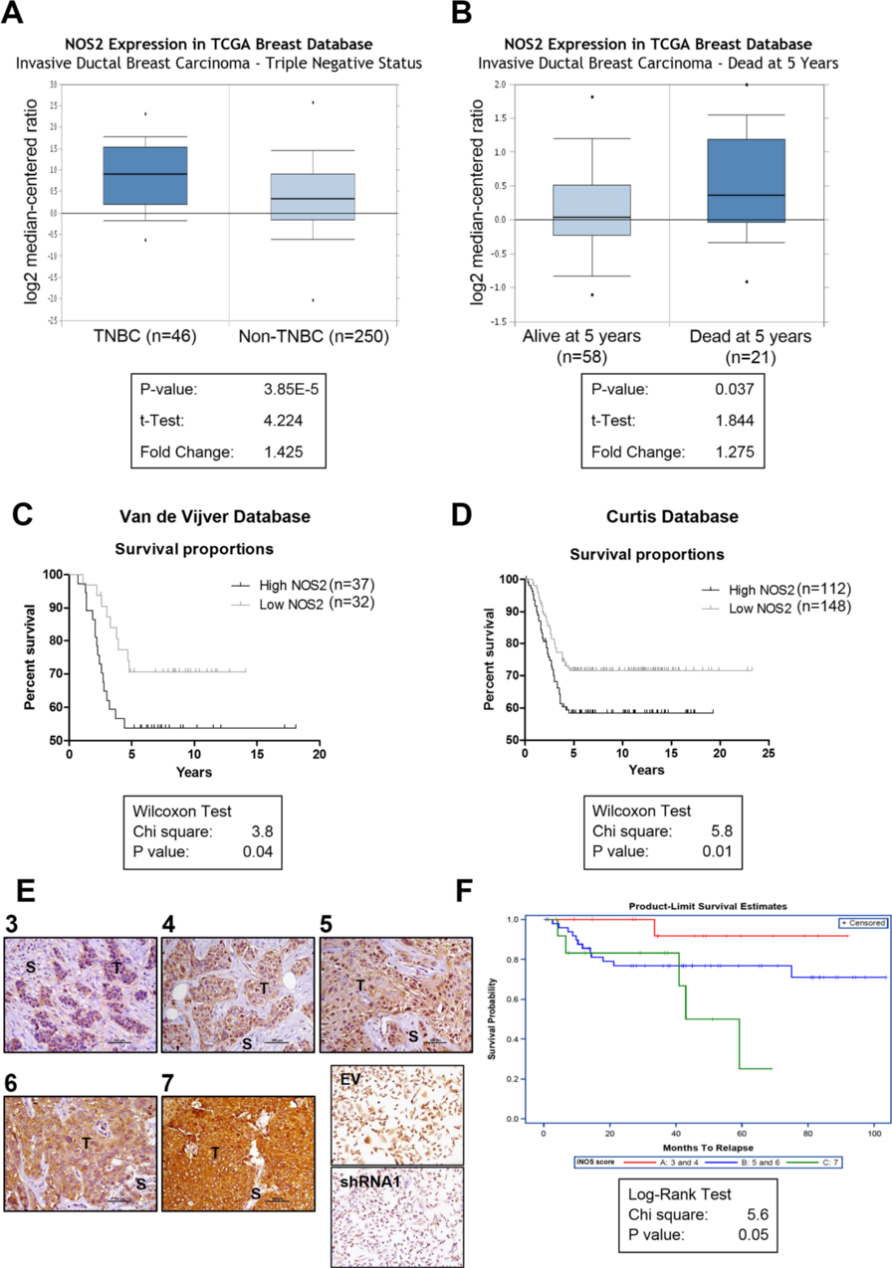
**Enhanced nitric oxide synthase 2 (NOS2) expression correlates with poor patient survival in invasive triple-negative breast cancer (TNBC). (A)** Higher *NOS2* mRNA expression in invasive TNBC versus non-TNBC (*P* = 3.85 × 10^−5^). **(B)** High *NOS2* expression correlates with death at 5 years in invasive breast carcinoma (*P* = 0.037). Kaplan-Meier survival analyses in **(C)** Van de Vijver (n = 69; *P* = 0.04) and **(D)** Curtis (n = 260; *P* = 0.01) breast databases show that high *NOS2* expression correlates with worse overall survival of patients with TNBC. **(E)** Immunohistochemical analysis of TNBC human samples for inducible nitric oxide synthase (iNOS) protein expression. Weak-to-moderate (3 or 4), moderate-to-strong (5 or 6), and strong (7) were the cutoffs established for further analysis of survival. Several samples showed expression in both tumor (T) and stromal (S) cells (original optical objective: 20×). MDA-MB-231 cells transfected with either *NOS2*-directed shRNA (shRNA1) or empty vector (EV) were used as negative and positive control of iNOS staining, respectively (original optical objective: 10×). Counterstain: hematoxylin. **(F)** Increased iNOS expression is associated with less patient survival when compared with low iNOS expression. Kaplan-Meier survival analysis of TNBC human patient samples (n = 83) (*P* = 0.05). shRNA1, small hairpin RNA 1; TCGA, The Cancer Genome Atlas.

iNOS has been described to be mediator of metastasis in different cancer types [20,21]. Elevated iNOS expression has been linked to poor survival in patients with ERα-negative breast cancer [7]. We hypothesized that enhanced iNOS expression in TNBC correlates with poor patient survival and metastases. Oncomine Cancer Microarray database analysis of *NOS2* expression in TCGA breast database showed higher *NOS2* expression in invasive TNBC (n = 46) versus non-TNBC (n = 250) (fold change 1.425, *P* = 3.85 × 10^−5^) (Figure 1A). Patient survival analysis demonstrated a correlation between increased *NOS2* expression and worse survival at 5 years in patients with invasive ductal breast carcinoma (n = 79) (fold change 1.275, *P* = 0.037) (Figure 1B). We further examined whether *NOS2* expression correlates with worse survival in two additional databases of TNBC. Analysis of Van de Vijver (n = 69 samples) [17] and Curtis (n = 260 samples) [18] databases confirmed that high *NOS2* expression was associated with poor survival in patients with TNBC (Figure 1C and D).

Next, we examined iNOS protein expression by immunohistochemistry in 83 surgically resected TNBC primary breast cancer samples, and correlated expression with known patient outcome. iNOS was primarily cytoplasmic, but some cells exhibited both cytoplasmic and nuclear localization (Figure 1E). Overall score showed that iNOS levels were weak to moderate (score 3 or 4) in 14 samples (16.9%) (Figure 1E, score 3 or 4), moderate to strong (score 5 or 6) in 50 samples (60.2%) (Figure 1E), and strong (score 7) in 19 specimens (22.9%) (Figure 1E). This stratification was used to analyze the correlation of iNOS expression and patient survival by using the Kaplan-Meier analysis. We confirmed that enhanced iNOS protein levels were associated with worse patient survival when compared with low iNOS expression (*P* = 0.05) (Figure 1F). These results demonstrate that increased iNOS by mRNA and protein expression in invasive TNBC is associated with poor patient survival.

### Inhibition of iNOS decreases cell proliferation, migration, and mammosphere formation of TNBC cells

**Figure 2:**
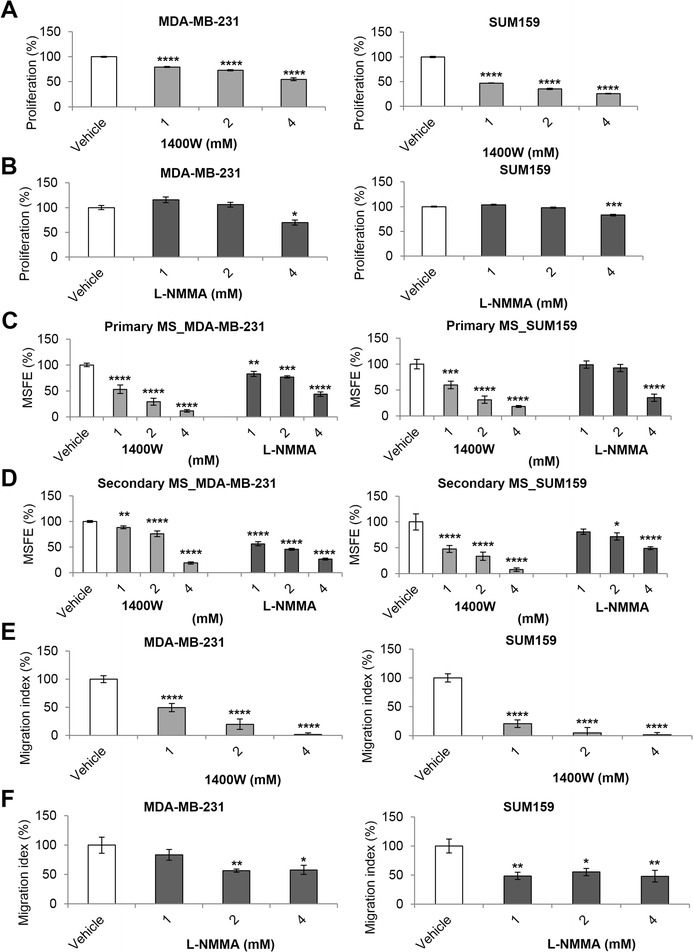
**Effects of inducible nitric oxide synthase (iNOS) inhibitors on tumor cell proliferation, migration, and mammosphere formation in triple-negative breast cancer (TNBC) cell lines.** Proliferation **(A, B)** and primary **(C)** and **(D)** secondary mammosphere, and migration index of MDA-MB-231 and SUM159 cell lines treated with 1400 W **(E)** and L-NMMA **(F)** for 96 hours. Results were normalized to vehicle. Data are presented as mean ± standard error of the mean. *P <0.05, **P <0.01, ***P <0.001, ****P <0.0001. 1400 W, N-[[3-(aminomethyl)phenyl]methyl]-ethanimidamide; L-NMMA, N^G^-monomethyl-L-arginine; MSFE, mammosphere-forming efficiency.

**Figure 3:**
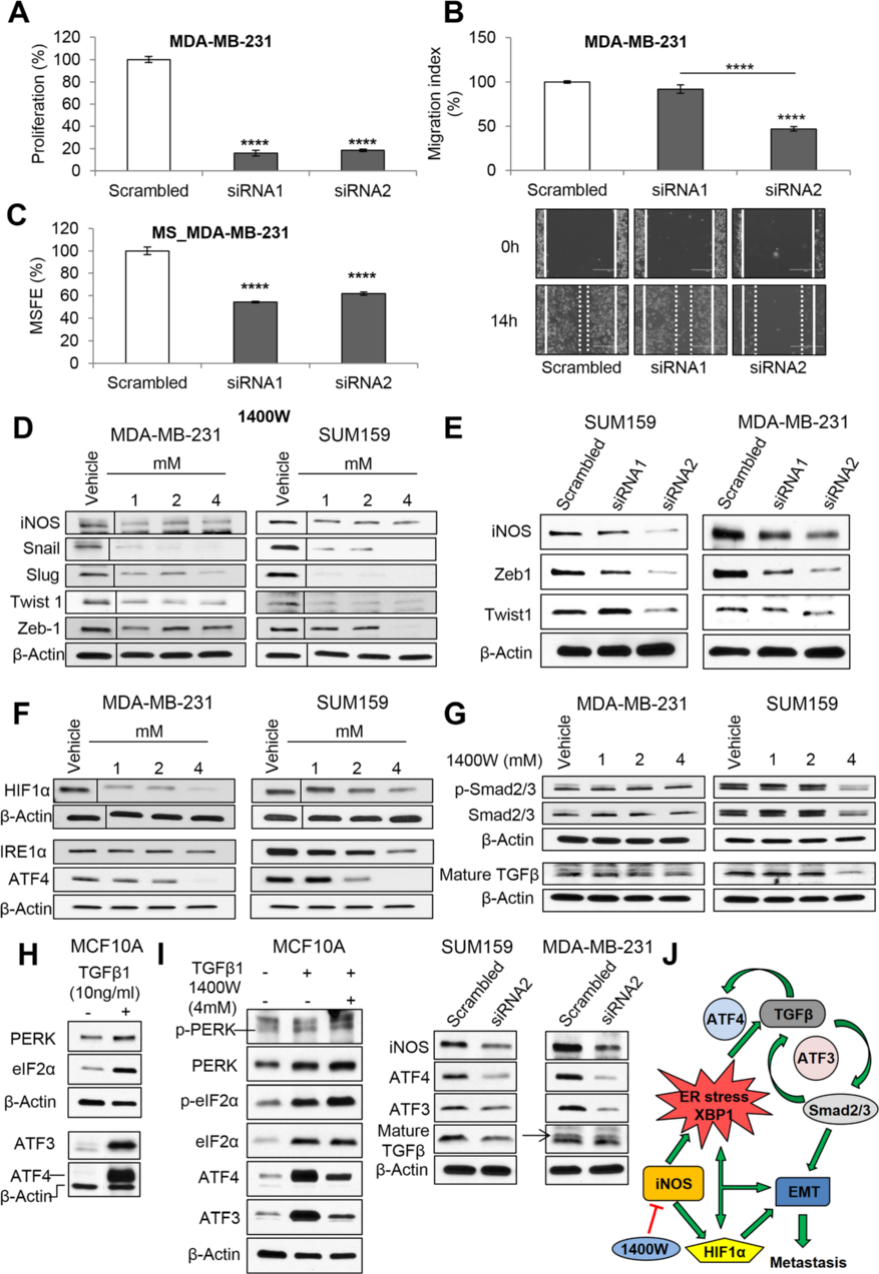
**iNOS knockdown impairs tumorigenicity and EMT by a dual impact on HIF1α and ER stress/TGFβ/AFT4/ATF3 crosstalk.** Proliferation **(A)**, migration **(B)**, and mammosphere-forming efficiency (MSFE) **(C)** in MDA-MB-231 cells transiently transfected with two different *NOS2*-directed siRNAs (siRNA1 and siRNA2) compared with scrambled control. Western blot analysis of NOS isoforms (iNOS, eNOS, and nNOS) and EMT transcription factors in MDA-MB-231 and SUM159 cell lines treated with **(D)** 1400 W and **(E)** siRNA-mediated *NOS2* knockdown. **(F)** Selective iNOS inhibition reduced hypoxia (HIF1α) and ER stress markers (IRE1α and ATF4). **(G)** Phospho-Smad2/3, Smad2/3, and mature TGFβ protein levels in MDA-MB-231 and SUM159 cells. **(H)** Recombinant TGFβ1 (10 ng/mL for 7 days) activates the PERK/eIF2α/ATF4/ATF3 axis in MCF10A. **(I)** Effects on the PERK/eIF2α/ATF4/ATF3 axis by co-treatment of recombinant TGFβ1 (10 ng/mL, 7 days) and 1400 W (4 mM) for 24 hours in MCF10A cells. iNOS, ATF4, ATF3, and mature TGFβ protein levels in siRNA-mediated *NOS2* knockdown (siRNA2) MCF10A cells for 96 hours. **(J)** Selective iNOS inhibition is postulated to impair EMT and tumor cell migration by an impact on HIF1α, ER stress (IRE1α/XBP1), and the crosstalk between ATF4, ATF3, and TGFβ. Results were normalized to scrambled. Data are presented as mean ± standard error of the mean. ****P <0.0001. 1400 W, N-[[3-(aminomethyl)phenyl]methyl]-ethanimidamide; ATF3, activating transcription factor 3; ATF4, activating transcription factor 4; EMT, epithelial-mesenchymal transition; ER, endoplasmic reticulum; HIF1α, hypoxia-inducible factor 1α; iNOS, inducible nitric oxide synthase; siRNA, small interfering RNA; TGFβ, transforming growth factor β.

High concentrations of 1400 W (1, 2, and 4 mM) significantly decreased proliferation in both cell lines (Figure 2A). Similar results were observed for L-NAME (Additional file 1: Figure S1A). L-NMMA at the highest concentration (4 mM) showed anti-proliferative activity in both cell lines (Figure 2B). Resistance to treatment and metastasis may arise from a subpopulation of CSCs within a heterogeneous primary cancer [22,23]. iNOS inhibition decreased primary MSs in both cell lines (Figure 2C; Additional file 1: Figure S1B; Additional file 2: Figure S2A; and Additional file 3: Figure S3A). We found reduced secondary MS in both cell lines for all the inhibitors tested (Figure 2D; Additional file 1: Figure S1C; Additional file 2: Figures S2B; and Additional file 3: Figure S3B). We show enhanced iNOS expression in invasive TNBC (Figure 1A); we then investigated the role of iNOS in cell migration. Selective iNOS inhibition with 1400 W caused a marked dose-dependent decrease in migration in both cell lines (Figure 2E and Additional file 1: Figure S1D). L-NMMA-treated cells showed reduction in migration capacity (Figure 2F and Additional file 1: Figure S1E). Similar results were found for L-NAME (Additional file 4: Figure S4A). These results were further confirmed in siRNA-mediated iNOS (*NOS2*) knockdown MDA-MB-231 (Figure 3A-C) and SUM159 cells (Additional file 5: Figure S5A, B, and C). Collectively, the results indicate that basal levels of iNOS have a major role in CSC self-renewal and migrating properties of TNBC cell lines and a less pronounced effect on proliferation.

### Suppression of endogenous iNOS could impair EMT and cell migration by impairing HIF1α and the endoplasmic reticulum stress/TGFβ/AFT4/ATF3 crosstalk

EMT is evoked during tumor invasion and metastasis [3,24]. We examined the impact on NOS isoforms (iNOS, eNOS, and nNOS) after either selective or pan-inhibition (Figure 3D and Additional file 4: Figure S4B, C, and F). Selective iNOS blockade with 1400 W caused a reduction in protein levels of the EMT transcription factors Snail, Slug, and Twist1 (Figure 3D and Additional file 4: Figure S4D). Zeb1 protein levels were decreased at millimolar concentrations (Figure 3D). Though less consistent, similar results were found for the pan-NOS inhibitors (Additional file 4: Figure S4E and F). iNOS knockdown with siRNA correlated with a decrease of Zeb1 and Twist1 protein levels in both cell lines (Figure 3E). Overall, these data suggest that selective iNOS inhibition efficiently decreases migration of TNBC cell lines, and this is consistently correlated with a decrease of EMT transcription factors.

Different pathways are responsible for inducing EMT and metastasis of tumor cells; among them, NO is a common denominator of HIF1α and endoplasmic reticulum (ER) stress [25-27]. Our findings indicate that selective iNOS inhibition resulted in a dose-dependent decrease in hypoxia (HIF1α) (Figure 3F and Additional file 4: Figure S4G) and the ER stress markers IRE1α/splicedXBP1 (Figure 3F and Additional file 5: Figure S5E) and ATF4 (activating transcription factor 4) in both cell lines (splicedXBP1 was not detected in SUM159 cells; data not shown) (Figure 3F). Functional protein-protein interaction analysis unveiled a link between iNOS and TGFβ1 (Additional file 5: Figure S5D). We confirmed that 1400 W is able to inhibit transforming growth factor β (TGFβ) signaling in the absence (Figure 3G) and presence of recombinant TGFβ1 (Additional file 5: Figure S5F) through an undetermined mechanism. Additional protein-protein interaction analyses showed an interaction between ATF4 and ATF3 (activating transcription factor 3) (Additional file 5: Figure S5D), both activating transcription factors that interact with TGFβ [28,29]. We confirmed the crosstalk between ER stress through ATF4/ATF3 and TGFβ (Additional file 5: Figure S5G); similarly, recombinant TGFβ1 induced the PERK/eIF2α/ATF4/ATF3 axis (Figure 3H). Our results show that co-treatment of the iNOS inhibitor 1400 W and recombinant TGFβ1 (pretreatment for 7 days) for 24 hours was able to inhibit the stimulation of ATF4 and ATF3 protein levels by TGFβ1 independently of the PERK/eIF2α pathway. This result was further confirmed in siRNA-mediated iNOS knockdown cells (Figure 3I). Overall, our data demonstrate that iNOS inhibition could impair EMT and tumor cell migration by impairing ER stress (IRE1α/XBP1) and the crosstalk between ATF4, ATF3, and TGFβ.

### iNOS inhibition reduces tumor growth and tumor-initiating capacity and prevents lung metastases in mouse models of TNBC

**Figure 4:**
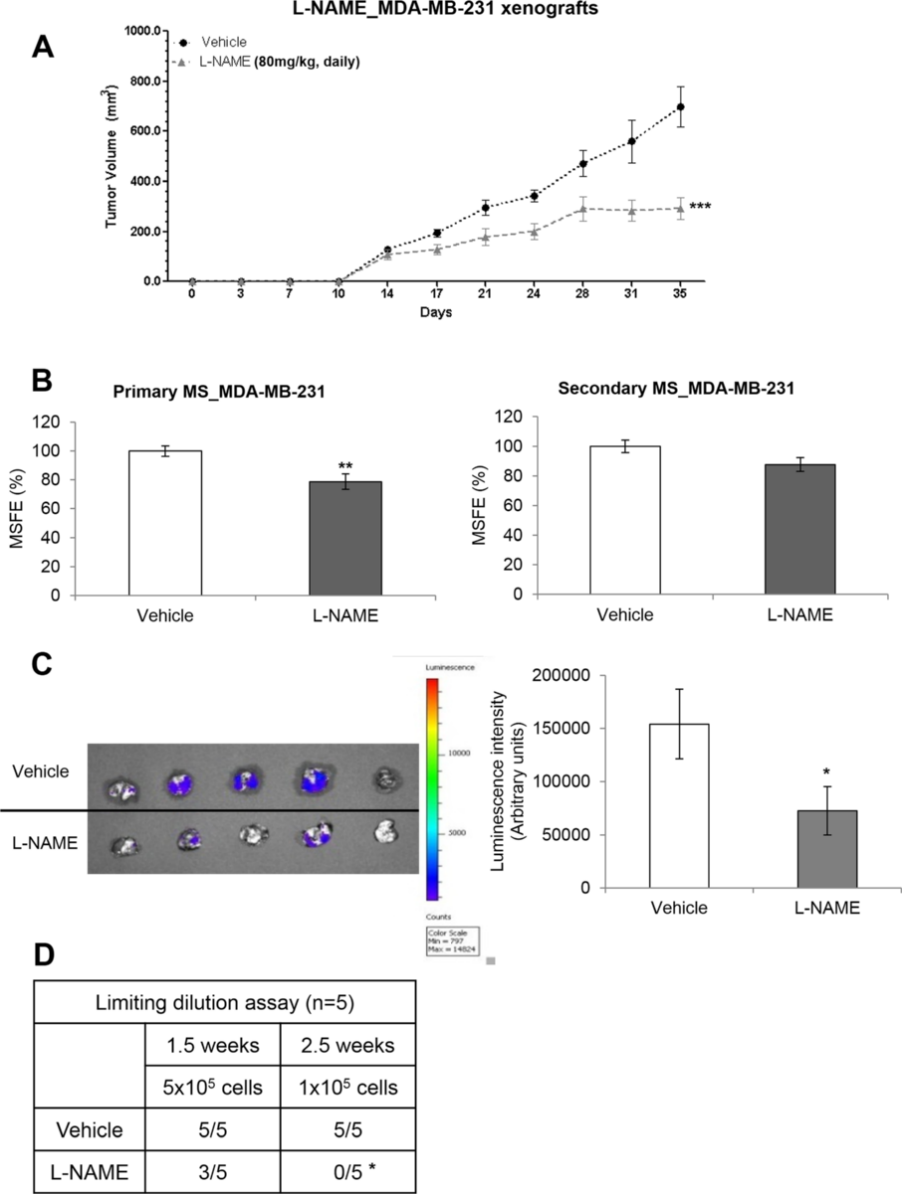
**Decrease in tumor initiation and lung metastases in MDA-MB-231 xenografts. (A)** Tumor volume of MDA-MB-231 breast xenografts (n = 5 per group) after daily injection of L-NAME (80 mg/kg, intraperitoneal). **(B)** Primary and secondary mammosphere of cancer cells isolated from tumor tissue. **(C)** Luminescence of MDA-MB-231 L/G tumor cells in lungs of vehicle- and L-NAME-treated mice. **(D)** Tumor-initiating capacity of tumor cells (limiting dilution assay). Results were normalized to vehicle. Data are presented as mean ± standard error of the mean. *P <0.05, **P <0.01, ***P <0.001. L-NAME, N5-[imino(nitroamino)methyl]-L-ornithine methyl ester; MSFE, mammosphere-forming efficiency.

Considering our *in vitro* data, we next investigated whether L-NAME was able to prevent tumor initiation and metastasis of MDA-MB-231 L/G cells in mice. L-NAME significantly reduced tumor growth (*P* = 0.001) (Figure 4A) as well as primary MS (Figure 4B). Additionally, tumor-initiating capacity of CSCs was assessed with LDA. All of the animals of the vehicle group developed tumors, but treatment with L-NAME yielded 3/5 tumors at 1.5 weeks with 5 × 10^5^ cells. The same results were observed in the vehicle group at 2.5 weeks with 1 × 10^5^ cells compared with the L-NAME-treated group (0/5 tumors) (*P* <0.05) (Figure 4D). *Ex vivo* imaging of lungs in the presence of luciferin showed higher fluorescence in the vehicle group compared with the L-NAME group (Figure 4C). These results suggest that iNOS inhibition with daily L-NAME may prevent metastasis to lungs in a TNBC mouse model.

To translate our results into future clinical trials, we used the pan-NOS inhibitor L-NMMA for further study. This is the first report using this drug re-purposed for this indication, with a paucity of data on the effective preclinical dose as an anti-cancer therapeutic. Thus, daily injections of 80 mg/kg L-NMMA were first given to SUM159 xenograft-bearing mice alone or in combination with docetaxel. After 10 days, no differential effect was observed between groups, and the daily dose was increased to 200 mg/kg. The tumor growth was efficiently blocked by L-NMMA administered alone or in combination with docetaxel (Additional file 6: Figure S6A). Higher proliferating rate (Ki67) in the vehicle and chemotherapy groups compared with the L-NMMA and combination groups (Additional file 6: Figure S6B and C) was observed. L-NMMA efficiently blocked the docetaxel-enhanced MSFE (Additional file 6: Figure S6D). LDA showed that the docetaxel and combination groups exhibited 12/12 and 6/12 tumors, respectively, at 7 weeks with 5 × 10^4^ cells. At week 9, we observed significant reduction in tumor-initiating capacity as different groups with 2 × 10^4^ cells yielded 3/12 and 0/12 tumors in docetaxel and combination, respectively (*P* <0.05) (Additional file 6: Figure S6E).

**Figure 5:**
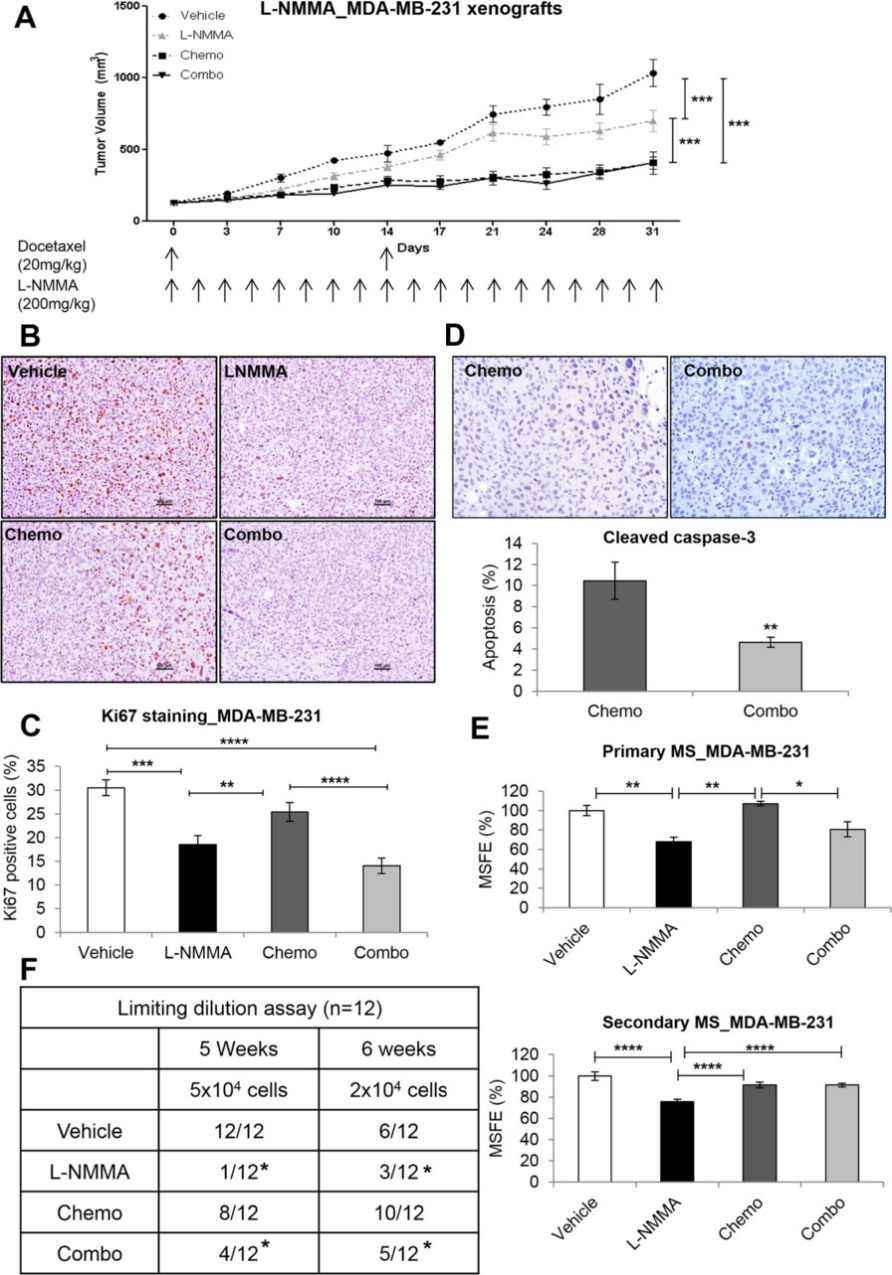
***In vivo*** **effects of L-NMMA in MDA-MB-231 xenografts. (A)** Tumor volume of MDA-MB-231 breast xenografts (n = 10 per group) treated with vehicle, L-NMMA, docetaxel, and combination. **(B)** Illustrative images of Ki67 staining in the vehicle, L-NMMA, docetaxel, and combination groups. Original optical objective: 10×. Counterstain: hematoxylin. **(C)** Cell proliferation of tumor xenografts with Ki67 immunostaining. **(D)** Nuclear cleaved caspase-3 staining in the chemo and combo groups. **(E)** Primary and secondary mammosphere of breast cancer cells isolated from tumor tissue. **(F)** Tumor-initiating capacity of tumor cells. Results were normalized to vehicle. For proliferation and apoptosis, 1,000 cells were counted from 10 different fields, and the percentage was determined. Data are presented as mean ± standard error of the mean. *P <0.05, **P <0.01, ***P <0.001, ****P <0.0001. L-NMMA, N^G^-monomethyl-L-arginine; MSFE, mammosphere-forming efficiency.

Similarly, docetaxel and L-NMMA (200 mg/kg, daily) were given to MDA-MB-231 xenograft-bearing mice. We found reduced tumor growth in the L-NMMA group compared with vehicle, but there was no change between the docetaxel and combination groups (Figure 5A). These results were further correlated with lower proliferation rate (Ki67) in the L-NMMA and combination groups (Figure 5B and C). Additionally, higher apoptosis levels in docetaxel-treated xenografts were found; these results might offset the high proliferation rate relative to the combination group (Figure 5D). A decrease in MS formation was seen for both L-NMMA and combination compared with vehicle and chemotherapy alone (Figure 5E). LDA showed that the vehicle-treated and docetaxel-treated groups presented 12/12 and 8/12 tumors, respectively, with a significant decrease in L-NMMA-treated (1/12) and combination-treated (4/12) xenografts at 5 weeks with 5 × 10^4^ cells. After 6 weeks, a decrease in both the L-NMMA and combination groups (3/12 and 5/12, respectively) compared with the vehicle and docetaxel groups (6/12 and 8/12, respectively) was observed (*P* <0.05) (Figure 5F). Our results in the MDA-MB-231 xenograft model do not show additional benefit over chemotherapy in terms of tumor volume; however, it is clear that the benefits of L-NMMA lie in the ability of this inhibitor to reduce self-renewal and tumor initiation capacities of CSCs.

Our investigations demonstrate that L-NMMA plasma levels are cleared rapidly (Additional file 7: Figure S7A), whereas it accumulates in the tumor tissue (Additional file 7: Figure S7B) and inhibits the conversion of L-arginine to L-citrulline and NO by iNOS (Additional file 7: Figure S7C) 24 hours after completion of treatment. This inhibition led to a decrease in total NO production as seen in SUM159 cells (Additional file 7: Figure S7D). Overall, these results demonstrate that *in vivo* iNOS inhibition with L-NMMA decreased tumor growth, cell proliferation, and tumor-initiating capacity of CSCs, together with a significant reduction in lung metastases.

### Efficient dose regimen of L-NMMA and docetaxel with potential clinical application

**Figure 6:**
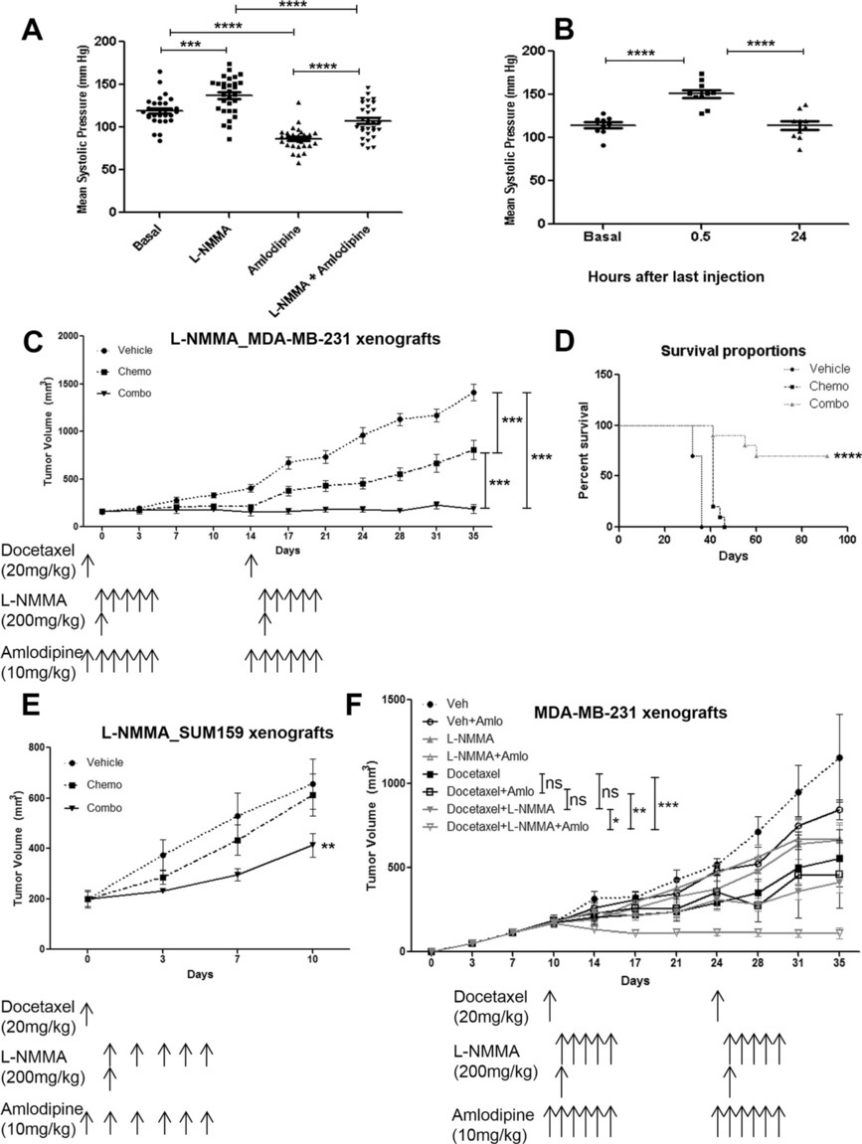
**Clinically relevant dose regimen of L-NMMA in orthotopic mouse models of triple-negative breast cancer. (A)** Mean systolic pressure of mice (n = 5) giving one cycle of the dose rate proposed in this study. **(B)** Mean systolic pressure of mice 30 minutes and 24 hours after the last injection of one cycle treatment (n = 5). **(C)** Tumor volume of MDA-MB-231 xenografts (n = 10 per group) treated with vehicle, docetaxel, and combination (docetaxel + amlodipine + L-NMMA). **(D)** Kaplan-Meier survival curve of vehicle-, chemotherapy-, and combo-treated MDA-MB-231 xenograft-bearing mice. **(E)** Tumor volume of SUM159 xenografts (n = 10 per group) treated with vehicle, docetaxel, and combination. **(F)** Effects of amlodipine on tumor volume in MDA-MB-231 xenografts (n = 5 per group). Data are presented as mean ± standard error of the mean. *P <0.05, **P <0.01, ***P <0.001, ****P <0.0001. L-NMMA, N^G^-monomethyl-L-arginine; ns, not significant.

Clinically, L-NMMA causes acute BP elevation through inhibition of constitutive eNOS [30]. Here, we propose a regimen with an attenuated duration of L-NMMA, at a dose comparable to that previously described clinically [31], together with the anti-hypertensive amlodipine and docetaxel. Oral L-NMMA significantly increased the mean systolic pressure compared with basal levels in mice, and this elevation was efficiently reversed by amlodipine (Figure 6A). This elevation in BP was transient and disappeared 24 hours after the last injection of L-NMMA (Figure 6B).

The combination of L-NMMA, amlodipine, and docetaxel was able to decrease tumor growth in an MDA-MB-231 orthotopic model (Figure 6C). This dose regimen also improved survival in comparison with the docetaxel-treated group (*P* = 0.0001) (Figure 6D). Similar results were found in SUM159 xenografts (Figure 6E). We tested the effect of amlodipine on tumor volume in MDA-MB-231 tumor-bearing mice (n = 5 per group). A significant reduction in tumor volume was observed in docetaxel + amlodipine + L-NMMA compared with docetaxel (*P* <0.001), docetaxel + amlodipine (*P* <0.01), and docetaxel + L-NMMA (*P* <0.05) (Figure 6F). Overall, these data show that the dose regimen proposed herein is effective in reducing tumor growth and may result in improved survival.

## Discussion

TNBC is an extremely aggressive and lethal form of cancer lacking effective targeted therapies. Patients with TNBC show higher risk of metastasis and tumor relapse [1,2]. We have recently described two novel cancer genes (*RPL39* and *MLF2*) that are important for tumor initiation and metastasis and are regulated by the NO signaling pathway [32]. iNOS predicts for worse survival in patients with basal-like ER-negative breast cancer and has been suggested to increase tumor aggressiveness by modulating CSCs as well as the metastatic propensity of cells [7]. This is the first report demonstrating that iNOS inhibition decreases tumorigenicity of TNBC cells by affecting cell proliferation, CSC self-renewal, and migration. Our *in vivo* experiments demonstrate the efficacy of a small-molecule iNOS inhibitor L-NMMA as a potential novel targeted therapy in patients with TNBC with immediate translation into human clinical trials.

We report that *NOS2* is commonly increased in invasive TNBC and is associated with poor survival of patients with invasive breast carcinoma. We provide additional data that high iNOS protein levels by immunohistochemistry in samples of patients with TNBC also correlate with worse outcome, consistent with earlier reports in ERα-negative [8] and invasive breast carcinoma [33]. Kaplan-Meier analysis of the Van de Vijver and Curtis databases as well as of the human TNBC patient samples strongly indicate that high iNOS expression is associated with poor overall survival in patients with TNBC. These observations establish that increased iNOS expression may be a marker of poor prognosis in patients with breast cancer [6-9].

iNOS expression has been correlated with increased tumor grade and aggressiveness of breast cancer cells [7,9]. Here, we describe the effect of iNOS on CSC self-renewal, tumor initiation, and the migrating capacity of TNBC cells. The anti-tumor activity of iNOS inhibitors has been previously reported in oral [34], glioblastoma [13], and breast cancer [35-38] and is consistent with our findings. Increased iNOS expression has been described to contribute to resistance to conventional treatment by promoting tumor initiation in glioblastoma cells [13,38]. Additionally, iNOS may influence CSC self-renewal by modulating *CD44* and *c-Myc* in ERα-negative breast cancer [7]. We demonstrate for the first time that iNOS inhibition decreased CSC self-renewal and tumor initiation in TNBC.

NO may either promote or inhibit metastatic events depending on endogenous levels [5]. The role of NOS inhibitors on metastasis has been previously studied, but the underlying mechanisms remain unclear. An early study demonstrated that the pan-NOS inhibitor L-NAME may decrease tumor growth and lung metastasis in a murine breast cancer model (EMT-6 cells) [39]. Similarly, L-NAME inhibited the invasive and migrating potential of two metastatic mammary cell lines (C3L5 and C10) [40]. In a study with the metastatic human adenocarcinoma HRT-18 cells, the invasiveness was substantially decreased by daily treatment with 500 μM of the selective iNOS inhibitor 1400 W [41]. More recently, 1400 W was shown to markedly inhibit spontaneous lung metastasis in a mouse model of adenoid cystic carcinoma of the oral cavity [34]. Our present results demonstrate that iNOS inhibition decreases cell migration and lung metastases in TNBC. It has been suggested that NO and iNOS may lead to early metastasis by inducing *IL-8* [7] and the CXC chemokine receptor 4 [42]. CSCs display mesenchymal features [2], resulting in increased cell migration and metastasis. iNOS inhibition decreased CSC self-renewal and tumor initiation, thus indicating that inhibitors against this pathway could reverse the transition of tumor cells to a more mesenchymal-like phenotype. Consistent with the effect on cell migration, selective iNOS inhibition and *NOS2* knockdown decreased transcription factors driving EMT in all of the TNBC cell lines tested.

EMT may be promoted by different signal transduction pathways like TGFβ, Wnt/β-catenin, Notch, and Hedgehog and multiple growth factors. EMT transcription factors (Snail, Slug, Twist1, or Zeb1) are activated by diverse intermediate effectors like c-Myc, Ets, HIF1α, or NFκB [43]. Additionally, ER stress has been linked to EMT in thyroid, alveolar epithelial, and human renal proximal tubule cells through activation of PERK, XBP1, or Grp78 [27,44-46]. Interestingly, among these disparate signaling networks, iNOS is the common denominator between HIF1α and ER stress [25-27]. Inhibition of endogenous iNOS-derived NO production was able to reduce HIF1α stabilization and protein levels in colon carcinoma cells [47]. Transcription factors Twist1, Snail, Slug, and Zeb1, among others, are directly or indirectly influenced by HIF1α [26]. Additionally, hypoxia induces ER stress and unfolded protein response and was recently linked to migration and sphere formation in breast cancer cells by activation of the PERK/ATF4/LAMP3 arm under hypoxic conditions [48]. Our results suggest that iNOS inhibition correlates with an impairment of TGFβ signaling via the ER stress ATF4/ATF3 axis. It is known that TGFβ stimulates ATF4 protein levels to suppress differentiation in calvarial osteoblasts [29]. Certain conditions such as ER stress through the PERK/eIF2α axis may activate ATF4, which in turn induces ATF3 transcription [49], whereas ATF3 itself is an activating transcription factor that enhances TGFβ, MS formation, and EMT in cooperation with Twist1 [28].

To determine a safe and effective regimen with clinical applicability was the main challenge of these preclinical studies. L-NMMA is a pan-NOS inhibitor that has been extensively studied in several clinical trials of circulatory shock [30]. In the cardiogenic shock trial, L-NMMA was safe and had few adverse events other than transient reversible hypertension [31]. In normotensive patients, L-NMMA was administered to patients with metastatic renal cell carcinoma prior to infusion of IL-2 [31]. Doses of 3 and 6 mg/kg did not induce clinically apparent side effects, and BP remained unchanged. At a dose level of 12 mg/kg, patients experienced an increase in systolic BP up to 25 mm Hg, without any clinical symptoms, which normalized rapidly on stopping the L-NMMA infusion. The dose rate in the present study was chosen, with modifications, on the basis of a previous clinical trial in patients with septic shock [31]. Our results demonstrate that tumor growth can be restrained by an attenuated regimen of 5 days of L-NMMA after chemotherapy, given together with amlodipine. López *et al*. [50] carried out a randomized, placebo-controlled, double-blind study of L-NMMA in patients with septic shock up to a maximum of 14 days. The regimen followed consisted of an initial dose of 2.5 mg/kg per hour and then was adjusted at different rates (0.5, 1, 2.5, 5, 7.5, 10, 15, and 20 mg/kg per hour). The current proposed dose regimen to be clinically tested as a novel anti-cancer therapeutic is at much lower total doses, which have been described [50].

## Conclusions

Our study provides new knowledge about the correlation between enhanced endogenous iNOS expression and poor survival in patients with TNBC. We show that the targeted therapy with iNOS inhibitors is able to inhibit not only tumor cell proliferation but also CSC self-renewal and migration, reducing tumor growth, tumor initiation, and the number of lung metastases. We suggest that inhibition of metastatic events may be due to a reduction of EMT transcription factors by inhibition of HIF1α, ER stress (IRE1α/splicedXBP1), and the TGFβ/ATF4/ATF3 axis. Finally, we propose a targeted therapeutic regimen, which decreases tumor growth and enhances survival rate *in vivo*; clinical trials targeting this pathway in patients with TNBC are being planned.

## Additional files

Additional file 1:
**L-NAME, 1400 W, and L-NMMA (micromolar range) on proliferation, migration, and mammosphere formation of TNBC cell lines.** Proliferation **(A)** and primary **(B)** and secondary **(C)** mammospheres of MDA-MB-231 and SUM159 cell lines treated with L-NAME. Impact of 1400 W **(D)** and L-NMMA **(E)** at micromolar concentrations on the migration index in MDA-MB-231 and SUM159 cells. Results were normalized to vehicle. Data are presented as mean ± standard error of the mean. **P* <0.05, ***P* <0.01, ****P* <0.001, *****P* <0.0001. 1400 W, N-[[3-(aminomethyl)phenyl]methyl]-ethanimidamide; L-NMMA, N^G^-monomethyl-L-arginine; TNBC, triple-negative breast cancer. Additional file 2:
**Representative images of mammospheres in MDA-MB-231 cells treated with iNOS inhibitors.** Illustrative images of primary **(A)** and secondary **(B)** mammospheres after treatment with 1400 W, L-NMMA (vehicle, 1, 2, 4 mM), and L-NAME (vehicle, 1, 2, 5 mM) for 96 hours. 1400 W, N-[[3-(aminomethyl)phenyl]methyl]-ethanimidamide; L-NAME, N5-[imino(nitroamino)methyl]-L-ornithine methyl ester; L-NMMA, N^G^-monomethyl-L-arginine; iNOS, inducible nitric oxide synthase. Additional file 3:
**Representative images of mammospheres in SUM159 cells treated with iNOS inhibitors.** Illustrative images of primary **(A)** and secondary **(B)** mammospheres after treatment with 1400 W, L-NMMA (vehicle, 1, 2, 4 mM), and L-NAME (vehicle, 1, 2, 5 mM) for 96 hours. 1400 W, N-[[3-(aminomethyl)phenyl]methyl]-ethanimidamide; L-NAME, N5-[imino(nitroamino)methyl]-L-ornithine methyl ester; L-NMMA, N^G^-monomethyl-L-arginine; iNOS, inducible nitric oxide synthase. Additional file 4:
**Migration and Western blot of NOS isoforms, EMT transcription factors, and hypoxia in TNBC cell lines treated with iNOS inhibitors.** **(A)** Tumor cell migration after treatment with L-NAME in MDA-MB-231 and SUM159 cell lines. **(B, C)** Western blot analysis of NOS isoforms (iNOS, eNOS, and nNOS) in MDA-MB-231 and SUM159 cells treated with 1400 W and L-NMMA. **(D, E)** EMT markers protein levels in MDA-MB-231 and SUM159 cells after treatment with micromolar concentrations of 1400 W or L-NMMA. **(F)** Western blot analysis of NOS isoforms and the EMT transcription factors in MDA-MB-231 and SUM159 cell lines treated with L-NAME. **(G)** Quantification of HIF1α protein levels relative to β-actin in MDA-MB-231 and SUM159 cells treated with 1400 W. Results were normalized to vehicle. Data are presented as mean ± standard error of the mean. **P* <0.05, ***P* <0.01. 1400 W, N-[[3-(aminomethyl)phenyl]methyl]-ethanimidamide; EMT, epithelial-mesenchymal transition; eNOS, endothelial nitric oxide synthase; HIF1α, hypoxia-inducible factor 1α; iNOS, inducible nitric oxide synthase; L-NAME, N5-[imino(nitroamino)methyl]-L-ornithine methyl ester; L-NMMA, N^G^-monomethyl-L-arginine; iNOS, inducible nitric oxide synthase; nNOS, neuronal nitric oxide synthase; TNBC, triple-negative breast cancer. Additional file 5:
**NOS2 knockdown decreases cell proliferation, migration, mammosphere formation, spliced XBP1, and Smad2/3 signaling.** Crosstalk between ER stress and TGFβ is shown. Proliferation **(A)**, migration **(B)**, and mammospheres **(C)** of SUM159 cells transiently transfected with two different NOS2-directed siRNAs (siRNA1 and siRNA2) compared with scrambled control. **(D)** Protein-protein interaction analysis (STRING 9.1) deciphered a link between NOS2, TGFβ1, and ATF4/ATF3 axis. **(E)** Unspliced *XBP1* (uXBP1), spliced *XBP1* (sXBP1), and β-actin RT-PCR cDNA amplicons from MDA-MB-231 cells treated with 1400 W for 96 hours. **(F)** The iNOS inhibitor 1400 W is able to reduce the Smad2/3 signaling in MDA-MB-231 cells under treatment with recombinant TGFβ1 (10 ng/mL) for 72 hours. **(G)** Tunicamycin (5 μM) confirmed the crosstalk between ER stress and TGFβ through ATF4/ATF3 transcription factors. Results were normalized to scrambled control. Data are presented as mean ± standard error of the mean. **P* <0.05, ***P* <0.01, ****P* <0.001, *****P* <0.0001. 1400 W, N-[[3-(aminomethyl)phenyl]methyl]-ethanimidamide; ATF3, activating transcription factor 3; ATF4, activating transcription factor 4; ER, endoplasmic reticulum; iNOS, inducible nitric oxide synthase; *NOS2*, nitric oxide synthase 2; RT-PCR, reverse transcription-polymerase chain reaction; shRNA, small hairpin RNA; siRNA, small interfering RNA; TGFβ, transforming growth factor β. Additional file 6:
***In vivo*** **effects of L-NMMA in SUM159 xenografts.** **(A)** Tumor volume of SUM159 breast xenografts (n = 10 per group) treated with vehicle, L-NMMA, chemotherapy, and combination. **(B)** Illustrative images of Ki67 staining in vehicle, L-NMMA, chemotherapy (docetaxel), and combination groups. Original optical objective: 10×. Counterstain: hematoxylin. **(C)** Cell proliferation of tumor xenografts is depicted as Ki67-positive cells. Cells (1,000) were counted from 10 different fields, and the percentage was determined. **(D)** Primary and secondary mammospheres of breast cancer cells isolated from tumor tissue. **(E)** Tumor-initiating capacity of tumor cells assayed by the limiting dilution method. Results were normalized to vehicle. Data are presented as mean ± standard error of the mean. **P* <0.05, ***P* <0.01, ****P* <0.001, *****P* <0.0001. L-NMMA, N^G^-monomethyl-L-arginine. Additional file 7:
**L-NMMA levels in plasma and tumor tissue.** **(A, B)** Ratiometric quantification of methylarginine in plasma and tumor tissue (MDA-MB-231 and SUM159 xenografts) by liquid chromatography-tandem mass spectrometry (LC-MS/MS). **(C)** iNOS catalyzes the reaction of L-arginine to L-citrulline + nitric oxide (NO). Ratiometric quantification of citrulline in SUM159 xenograft tissue by LC-MS/MS. **(D)** Total NO production in SUM159 cells treated with L-NMMA and 1400 W (4 mM) for 0.5, 2, 6, and 24 hours. Results were normalized to vehicle. Data are presented as mean ± standard error of the mean. **P* <0.05, ***P* <0.01, ****P* <0.001, *****P* <0.0001. 1400 W, N-[[3-(aminomethyl)phenyl]methyl]-ethanimidamide; L-NMMA, N^G^-monomethyl-L-arginine; iNOS, inducible nitric oxide synthase.

## Abbreviations

1400 W: N-[[3-(aminomethyl)phenyl]methyl]-ethanimidamide
ANOVA: analysis of variance
ATF3: activating transcription factor 3
ATF4: activating transcription factor 4
BP: blood pressure
CSC: cancer stem cell
DMEM: Dulbecco’s modified Eagle’s medium
EMT: epithelial-mesenchymal transition
eNOS: endothelial nitric oxide synthase
ER: endoplasmic reticulum
ERα: estrogen receptor alpha
HIF1α: hypoxia-inducible factor 1α
IL-8: interleukin-8
iNOS: inducible nitric oxide synthase
i.p.: intraperitoneal
LDA: limiting dilution assay
L-NAME: N5-[imino(nitroamino)methyl]-L-ornithine methyl ester
L-NMMA: N^G^-monomethyl-L-arginine
MS: mammosphere
MSFE: mammosphere-forming efficiency
nNOS: neuronal nitric oxide synthase
NO: nitric oxide
NOS: nitric oxide synthase
PCR: polymerase chain reaction
SCID: severe combined immunodeficiency
shRNA: small hairpin RNA
siRNA: small interfering RNA
TCGA: The Cancer Genome Atlas
TGFβ: transforming growth factor β
TNBC: triple-negative breast cancer
