# Supplementary figures and images for: Inhibition of iNOS as a novel effective targeted therapy against triple-negative breast cancer
Source: Breast Cancer Res. 2015 Feb 22;17(1):25. doi: 10.1186/s13058-015-0527-x (PMC4384389; doi:10.1186/s13058-015-0527-x)

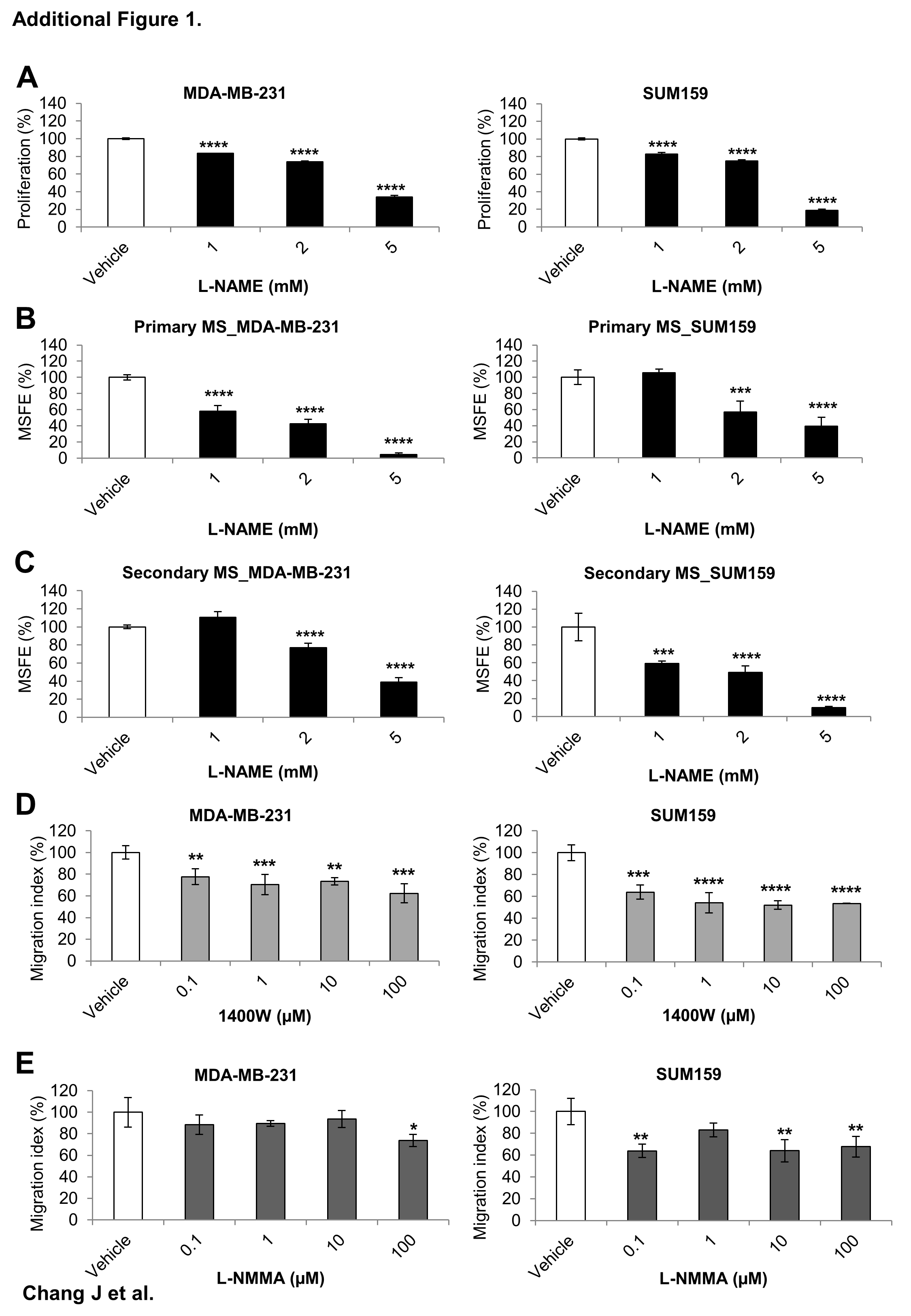

Supplement: Additional file 1: — L-NAME, 1400 W, and L-NMMA (micromolar range) on proliferation, migration, and mammosphere formation of TNBC cell lines. Proliferation (A) and primary (B) and secondary (C) mammospheres of MDA-MB-231 and SUM159 cell lines treated with L-NAME. Impact of 1400 W (D) and L-NMMA (E) at micromolar concentrations on the migration index in MDA-MB-231 and SUM159 cells. Results were normalized to vehicle. Data are presented as mean ± standard error of the mean. *P <0.05, **P <0.01, ***P <0.001, ****P <0.0001. 1400 W, N-[[3-(aminomethyl)phenyl]methyl]-ethanimidamide; L-NMMA, NG-monomethyl-L-arginine; TNBC, triple-negative breast cancer. [file 13058_2015_527_MOESM1_ESM.tif]

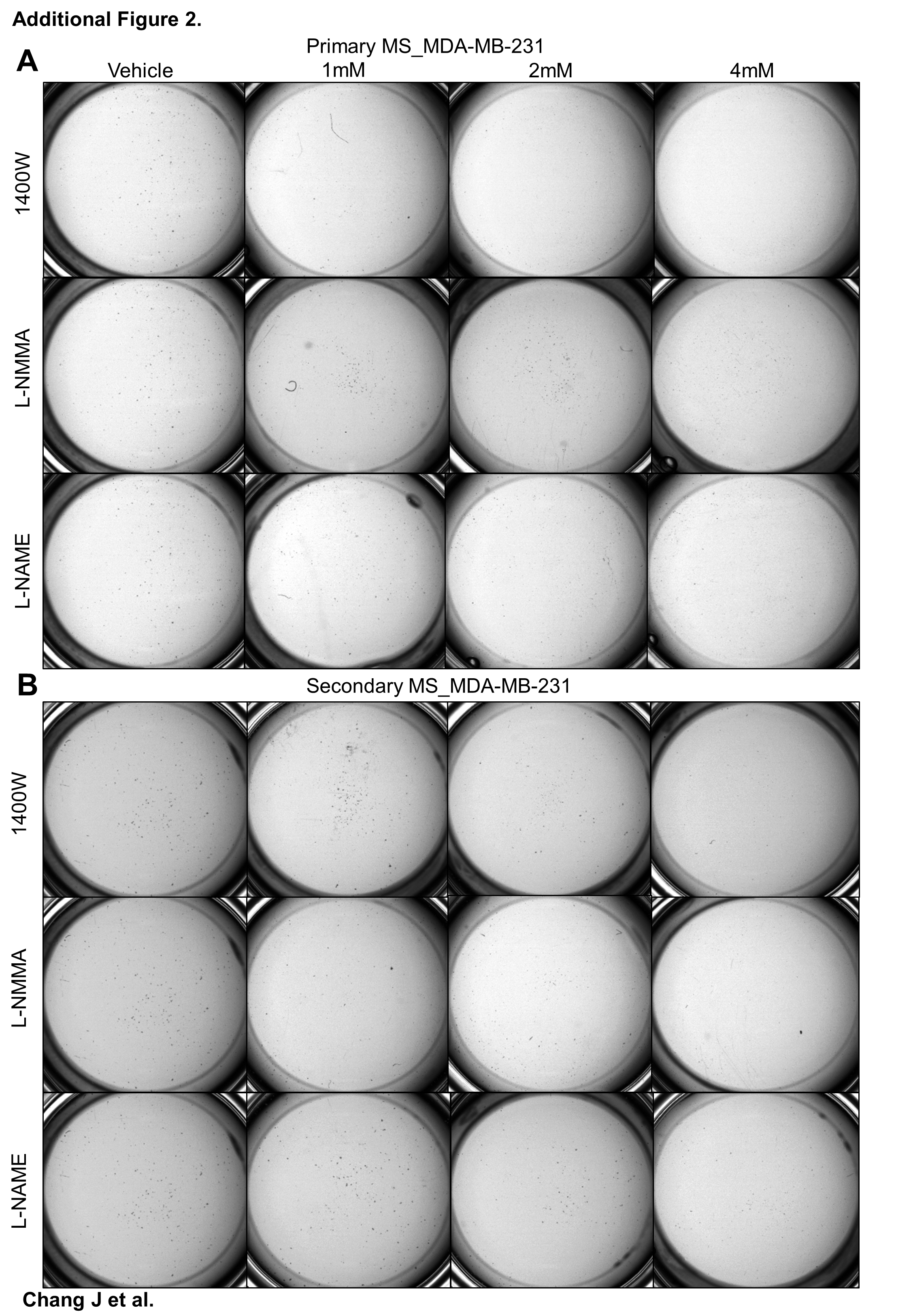

Supplement: Additional file 2: — Representative images of mammospheres in MDA-MB-231 cells treated with iNOS inhibitors. Illustrative images of primary (A) and secondary (B) mammospheres after treatment with 1400 W, L-NMMA (vehicle, 1, 2, 4 mM), and L-NAME (vehicle, 1, 2, 5 mM) for 96 hours. 1400 W, N-[[3-(aminomethyl)phenyl]methyl]-ethanimidamide; L-NAME, N5-[imino(nitroamino)methyl]-L-ornithine methyl ester; L-NMMA, NG-monomethyl-L-arginine; iNOS, inducible nitric oxide synthase. [file 13058_2015_527_MOESM2_ESM.tif]

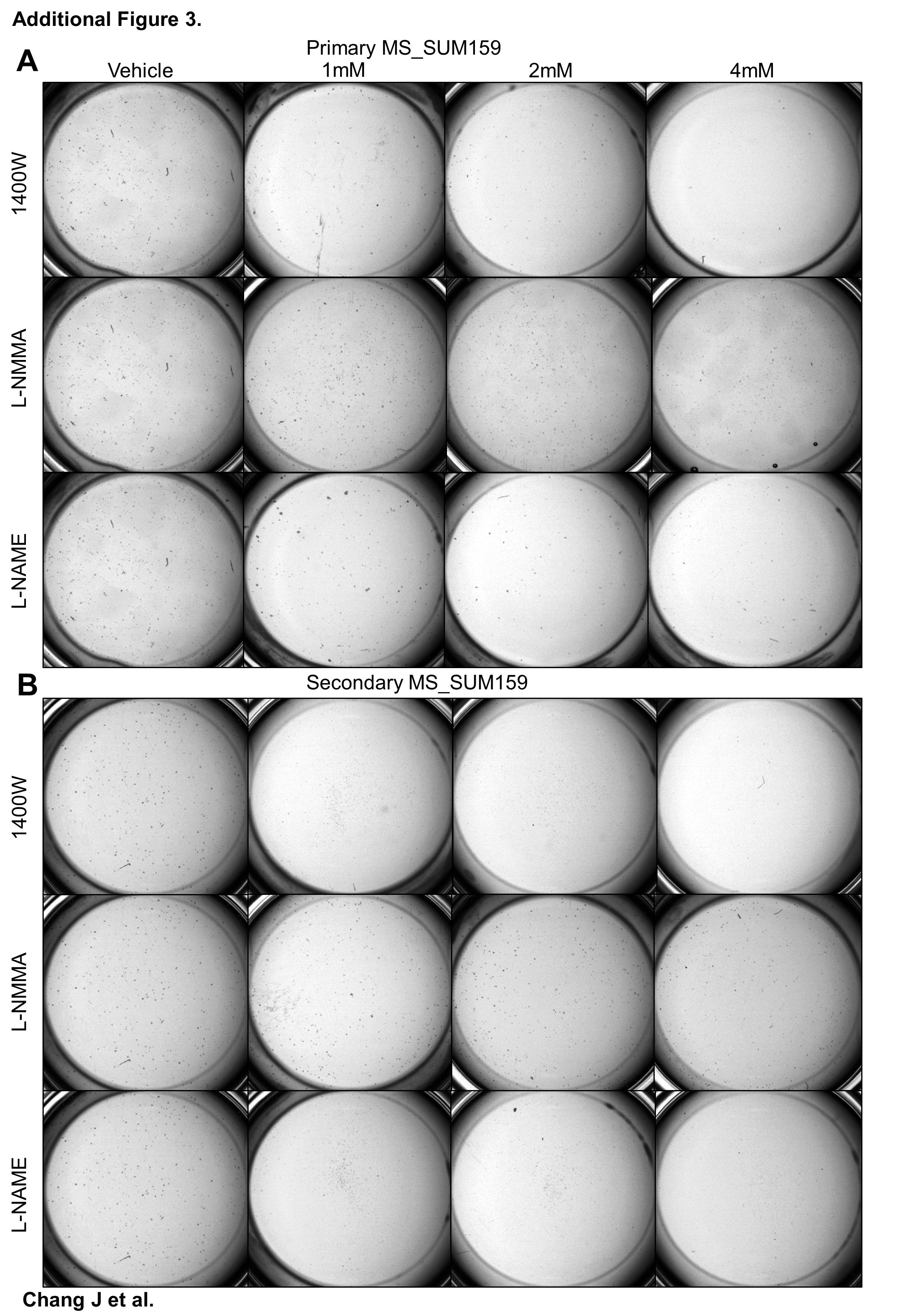

Supplement: Additional file 3: — Representative images of mammospheres in SUM159 cells treated with iNOS inhibitors. Illustrative images of primary (A) and secondary (B) mammospheres after treatment with 1400 W, L-NMMA (vehicle, 1, 2, 4 mM), and L-NAME (vehicle, 1, 2, 5 mM) for 96 hours. 1400 W, N-[[3-(aminomethyl)phenyl]methyl]-ethanimidamide; L-NAME, N5-[imino(nitroamino)methyl]-L-ornithine methyl ester; L-NMMA, NG-monomethyl-L-arginine; iNOS, inducible nitric oxide synthase. [file 13058_2015_527_MOESM3_ESM.tif]

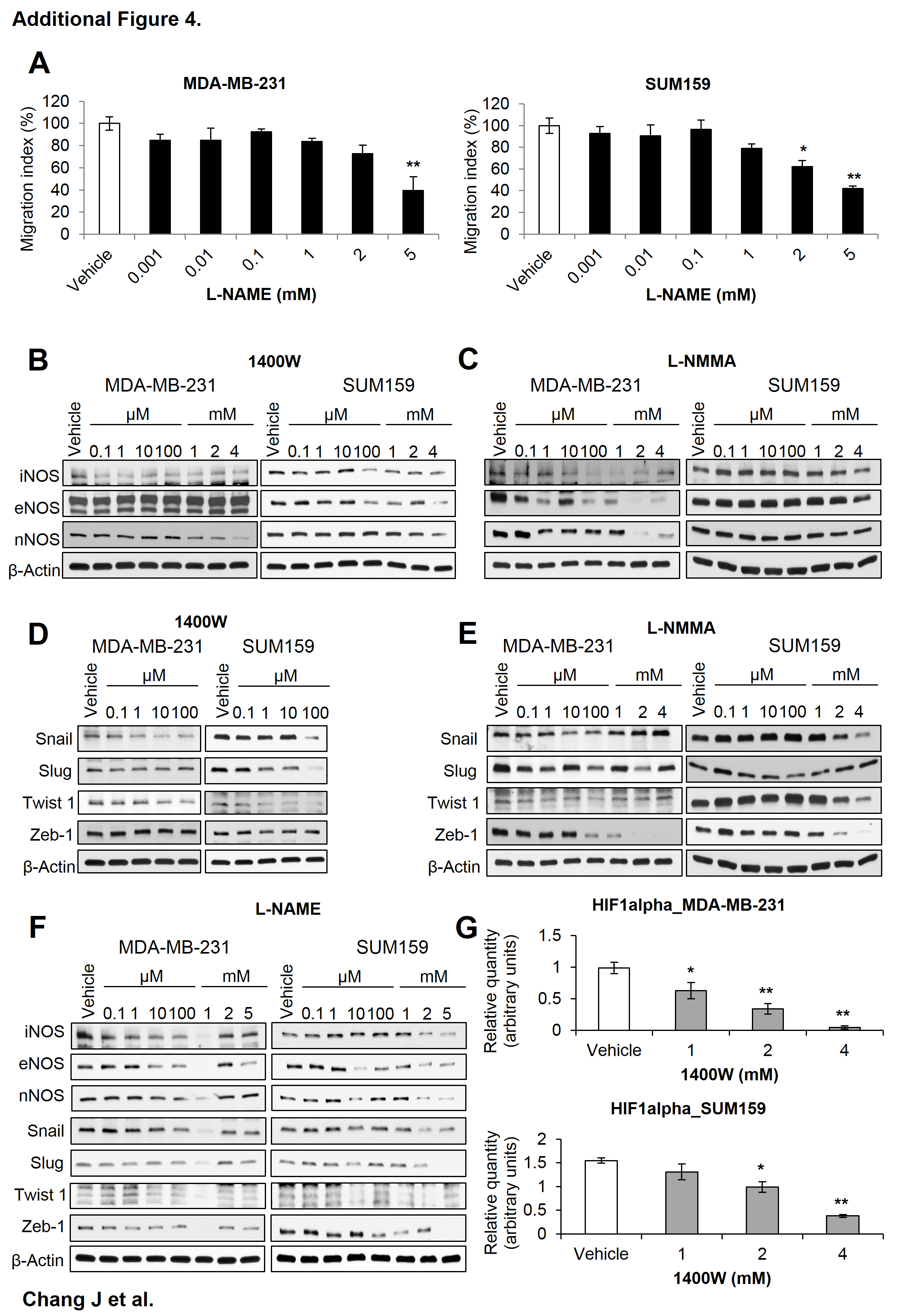

Supplement: Additional file 4: — Migration and Western blot of NOS isoforms, EMT transcription factors, and hypoxia in TNBC cell lines treated with iNOS inhibitors. (A) Tumor cell migration after treatment with L-NAME in MDA-MB-231 and SUM159 cell lines. (B, C) Western blot analysis of NOS isoforms (iNOS, eNOS, and nNOS) in MDA-MB-231 and SUM159 cells treated with 1400 W and L-NMMA. (D, E) EMT markers protein levels in MDA-MB-231 and SUM159 cells after treatment with micromolar concentrations of 1400 W or L-NMMA. (F) Western blot analysis of NOS isoforms and the EMT transcription factors in MDA-MB-231 and SUM159 cell lines treated with L-NAME. (G) Quantification of HIF1α protein levels relative to β-actin in MDA-MB-231 and SUM159 cells treated with 1400 W. Results were normalized to vehicle. Data are presented as mean ± standard error of the mean. *P <0.05, **P <0.01. 1400 W, N-[[3-(aminomethyl)phenyl]methyl]-ethanimidamide; EMT, epithelial-mesenchymal transition; eNOS, endothelial nitric oxide synthase; HIF1α, hypoxia-inducible factor 1α; iNOS, inducible nitric oxide synthase; L-NAME, N5-[imino(nitroamino)methyl]-L-ornithine methyl ester; L-NMMA, NG-monomethyl-L-arginine; iNOS, inducible nitric oxide synthase; nNOS, neuronal nitric oxide synthase; TNBC, triple-negative breast cancer. [file 13058_2015_527_MOESM4_ESM.tiff]

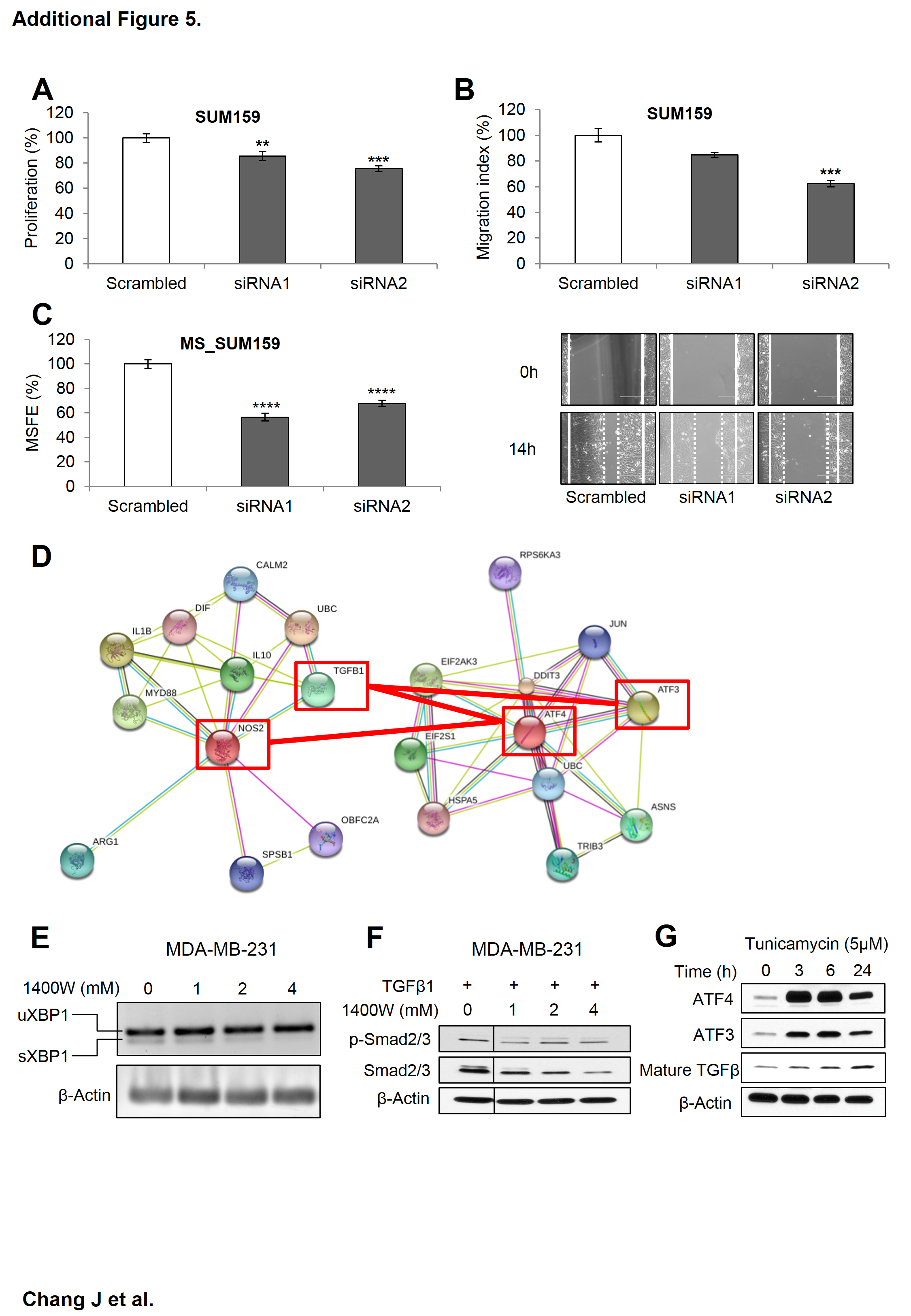

Supplement: Additional file 5: — NOS2 knockdown decreases cell proliferation, migration, mammosphere formation, spliced XBP1, and Smad2/3 signaling. Crosstalk between ER stress and TGFβ is shown. Proliferation (A), migration (B), and mammospheres (C) of SUM159 cells transiently transfected with two different NOS2-directed siRNAs (siRNA1 and siRNA2) compared with scrambled control. (D) Protein-protein interaction analysis (STRING 9.1) deciphered a link between NOS2, TGFβ1, and ATF4/ATF3 axis. (E) Unspliced XBP1 (uXBP1), spliced XBP1 (sXBP1), and β-actin RT-PCR cDNA amplicons from MDA-MB-231 cells treated with 1400 W for 96 hours. (F) The iNOS inhibitor 1400 W is able to reduce the Smad2/3 signaling in MDA-MB-231 cells under treatment with recombinant TGFβ1 (10 ng/mL) for 72 hours. (G) Tunicamycin (5 μM) confirmed the crosstalk between ER stress and TGFβ through ATF4/ATF3 transcription factors. Results were normalized to scrambled control. Data are presented as mean ± standard error of the mean. *P <0.05, **P <0.01, ***P <0.001, ****P <0.0001. 1400 W, N-[[3-(aminomethyl)phenyl]methyl]-ethanimidamide; ATF3, activating transcription factor 3; ATF4, activating transcription factor 4; ER, endoplasmic reticulum; iNOS, inducible nitric oxide synthase; NOS2, nitric oxide synthase 2; RT-PCR, reverse transcription-polymerase chain reaction; shRNA, small hairpin RNA; siRNA, small interfering RNA; TGFβ, transforming growth factor β. [file 13058_2015_527_MOESM5_ESM.tiff]

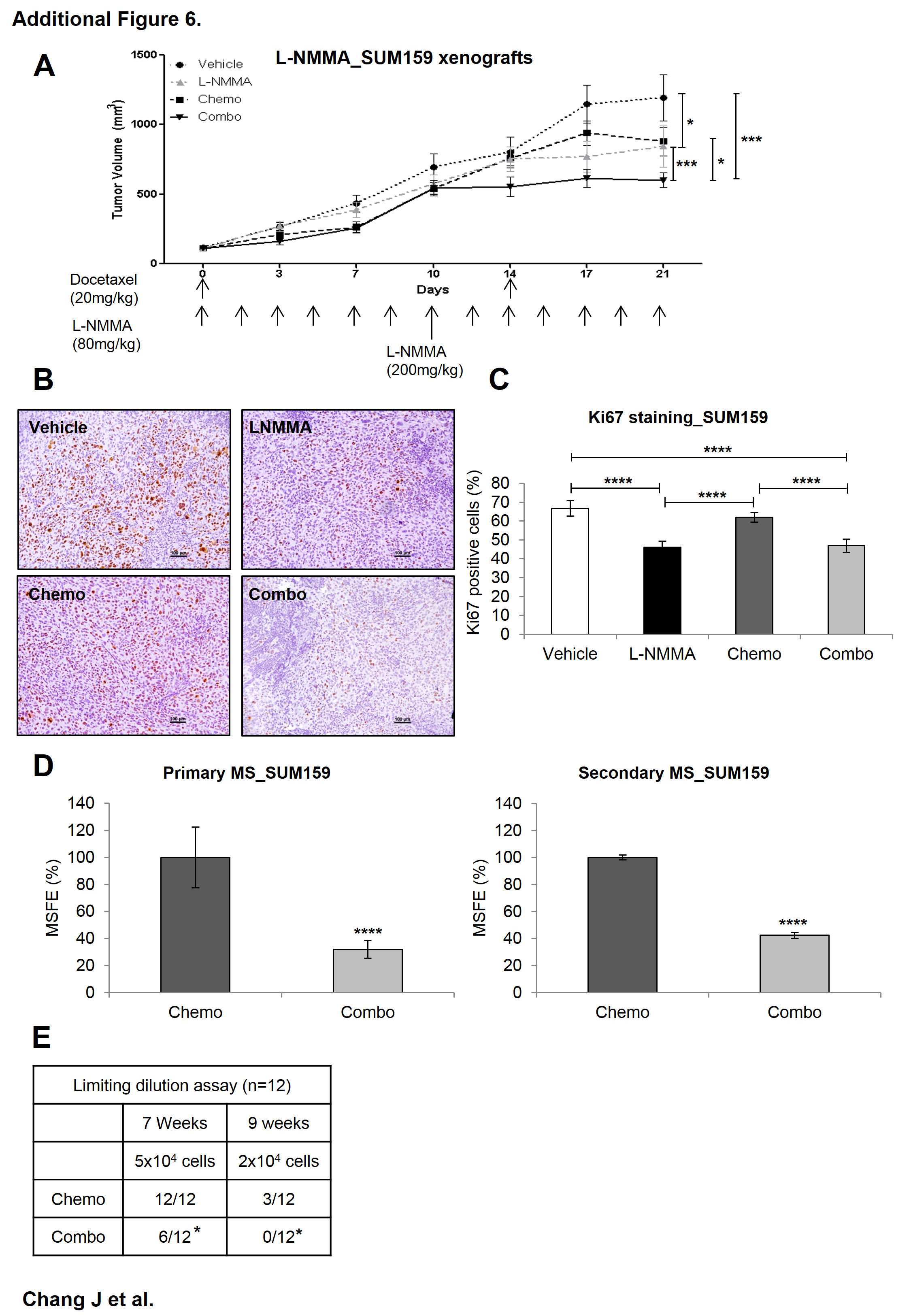

Supplement: Additional file 6: — In vivo effects of L-NMMA in SUM159 xenografts. (A) Tumor volume of SUM159 breast xenografts (n = 10 per group) treated with vehicle, L-NMMA, chemotherapy, and combination. (B) Illustrative images of Ki67 staining in vehicle, L-NMMA, chemotherapy (docetaxel), and combination groups. Original optical objective: 10×. Counterstain: hematoxylin. (C) Cell proliferation of tumor xenografts is depicted as Ki67-positive cells. Cells (1,000) were counted from 10 different fields, and the percentage was determined. (D) Primary and secondary mammospheres of breast cancer cells isolated from tumor tissue. (E) Tumor-initiating capacity of tumor cells assayed by the limiting dilution method. Results were normalized to vehicle. Data are presented as mean ± standard error of the mean. *P <0.05, **P <0.01, ***P <0.001, ****P <0.0001. L-NMMA, NG-monomethyl-L-arginine. [file 13058_2015_527_MOESM6_ESM.tiff]

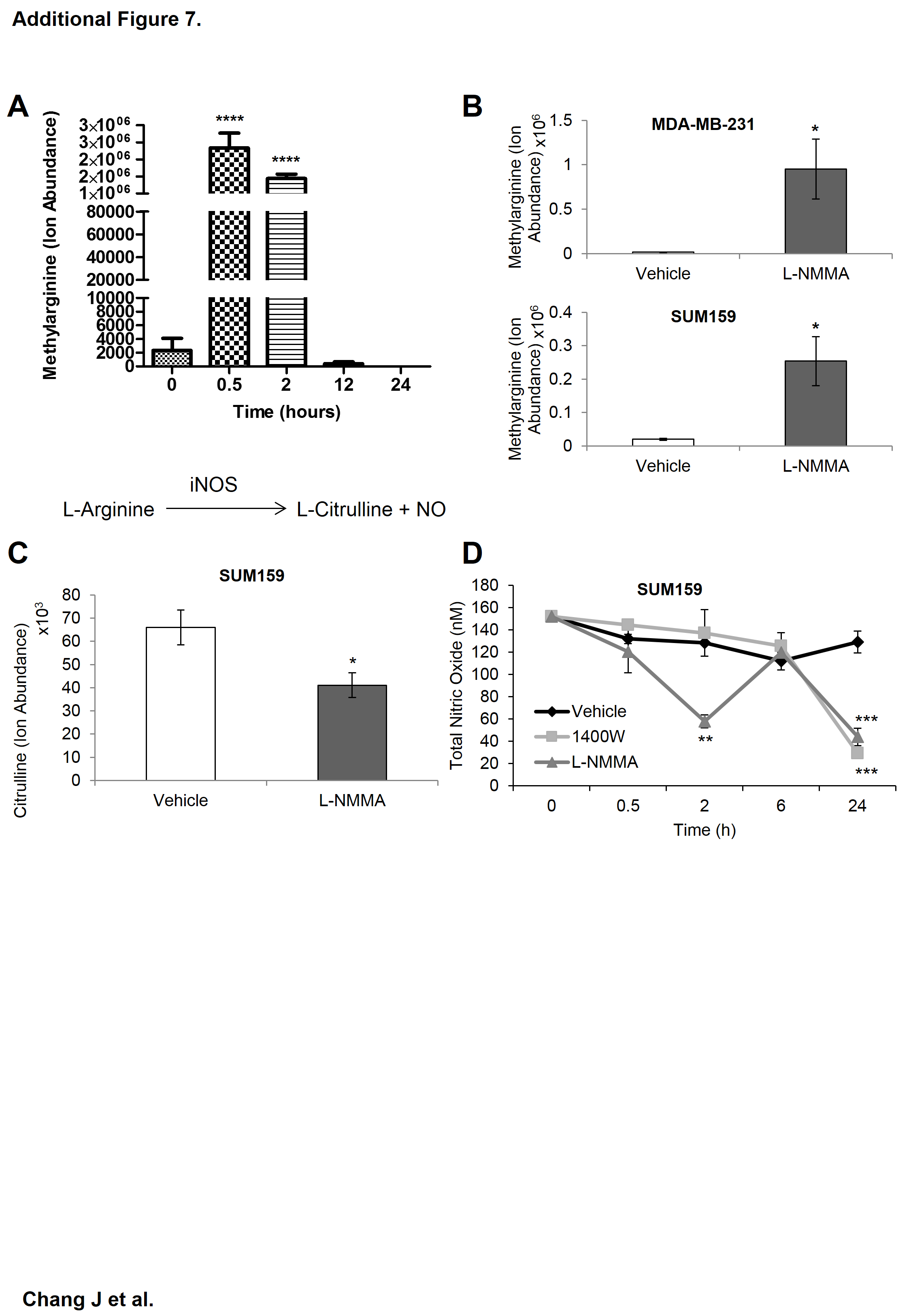

Supplement: Additional file 7: — L-NMMA levels in plasma and tumor tissue. (A, B) Ratiometric quantification of methylarginine in plasma and tumor tissue (MDA-MB-231 and SUM159 xenografts) by liquid chromatography-tandem mass spectrometry (LC-MS/MS). (C) iNOS catalyzes the reaction of L-arginine to L-citrulline + nitric oxide (NO). Ratiometric quantification of citrulline in SUM159 xenograft tissue by LC-MS/MS. (D) Total NO production in SUM159 cells treated with L-NMMA and 1400 W (4 mM) for 0.5, 2, 6, and 24 hours. Results were normalized to vehicle. Data are presented as mean ± standard error of the mean. *P <0.05, **P <0.01, ***P <0.001, ****P <0.0001. 1400 W, N-[[3-(aminomethyl)phenyl]methyl]-ethanimidamide; L-NMMA, NG-monomethyl-L-arginine; iNOS, inducible nitric oxide synthase. [file 13058_2015_527_MOESM7_ESM.tiff]
